# An Analysis Approach for High-Field fMRI Data from Awake Non-Human Primates

**DOI:** 10.1371/journal.pone.0029697

**Published:** 2012-01-06

**Authors:** Steffen Stoewer, Jozien Goense, Georgios A. Keliris, Andreas Bartels, Nikos K. Logothetis, John Duncan, Natasha Sigala

**Affiliations:** 1 Max Planck Institute for Biological Cybernetics, Tübingen, Germany; 2 Centre for Integrative Neuroscience, University of Tübingen, Tübingen, Germany; 3 Imaging Science and Biomedical Engineering, University of Manchester, Manchester, United Kingdom; 4 MRC Cognition and Brain Sciences Unit, Cambridge, United Kingdom; 5 Brighton and Sussex Medical School, University of Sussex, Brighton, United Kingdom; Cuban Neuroscience Center, Cuba

## Abstract

fMRI experiments with awake non-human primates (NHP) have seen a surge of applications in recent years. However, the standard fMRI analysis tools designed for human experiments are not optimal for analysis of NHP fMRI data collected at high fields. There are several reasons for this, including the trial-based nature of NHP experiments, with inter-trial periods being of no interest, and segmentation artefacts and distortions that may result from field changes due to movement. We demonstrate an approach that allows us to address some of these issues consisting of the following steps: 1) Trial-based experimental design. 2) Careful control of subject movement. 3) Computer-assisted selection of trials devoid of artefacts and animal motion. 4) Nonrigid between-trial and rigid within-trial realignment of concatenated data from temporally separated trials and sessions. 5) Linear interpolation of inter-trial intervals and high-pass filtering of temporally continuous data 6) Removal of interpolated data and reconcatenation of datasets before statistical analysis with SPM. We have implemented a software toolbox, fMRI Sandbox (http://code.google.com/p/fmri-sandbox/), for semi-automated application of these processing steps that interfaces with SPM software. Here, we demonstrate that our methodology provides significant improvements for the analysis of awake monkey fMRI data acquired at high-field. The method may also be useful for clinical applications with subjects that are unwilling or unable to remain motionless for the whole duration of a functional scan.

## Introduction

In human fMRI studies, there are theoretically and experimentally established preprocessing procedures that prepare the data for subsequent statistical analysis. First, all images of a session are spatially co-registered to either the first or the mean image of a time series to remove variance stemming from voxel position changes. Secondly, the realigned images are normalized to a template brain to prepare the data for later analysis at the group level. Third, images are spatially smoothed with a Gaussian kernel to reduce noise, and often to facilitate combination of data across subjects (see e.g. [1], [2] for detailed descriptions of each step). To suppress slowly varying trends and remove high-frequency noise, data are temporally filtered before running statistical tests.

However, using this conventional processing pipeline for awake non-human primate (NHP) data acquired at high magnetic field (7T) without specific adaptations is not appropriate for a variety of reasons, including the trial-based nature of NHP experiments, with inter-trial periods being of no interest, and the artefacts produced by field changes due to movement. In this paper we describe an approach to analysis of high-field data from NHPs, overcoming several limitations of standard automated processing packages.

One assumption underlying popular realignment algorithms, e.g. in Statistical Parametric Mapping (SPM, Wellcome Department of Imaging Neuroscience, London, UK. Accessed 2011 Dec 7), is a high degree of similarity and/or conservation of the subject's head shape across time within a session, with the possibility for shifts and rotations in any direction. In high-field awake NHP scans, this assumption is not met. The subject's head is not free to move, but is instead secured to the primate chair by an implanted head post . Thus, head motion in the conventional sense is not an issue. Instead, due to the sensitivity of the high field to animal jaw and body movements, other more complex issues arise [3].

**Figure 1:**
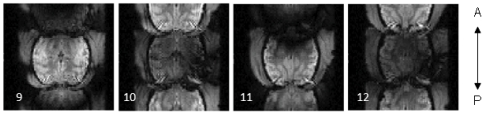
Ghosting artefact due to animal movement in segmented EPI -acquisition. Example of ghosting artefact in a 2-shot GE EPI image in four axial slices (9–12) from a volume acquired during a period of animal movement.

With increasing field strength, subject movement has a correspondingly larger impact on image quality. Because they significantly alter the B_0_ field at high field strength, subject movements during image acquisition cause substantial image distortions and signal changes, e.g. [4]. One important source of such distortions is jaw movement, inevitable since animals participate for juice reward. Swallowing also causes image warping [5], and artefacts can also be introduced by movements of the animal's body or limbs. An established procedure to detect and prevent such movements during experiments is training the animals to remain still during certain experiment phases (trials) with the help of motion sensors attached to the animal chair [3]. Large body or jaw movements lead to image degradation in the form of severe ghosting with segmented EPI sequences (for an example from our data see Figure 1). In addition, shifts of the brain in the phase-encoding direction can occur when there is a position change of the animal during the inter-trial phase. These shifts are not caused by actual head movements, but by changes in the B_0_ field induced by position changes of the animal's body. Other authors have addressed these issues with the help of field maps [6]–[8].

In the case of NHP imaging with very frequent body movements, field maps would have to be acquired between every trial, which would require special imaging sequences not available to most laboratories at present.

To address some of these problems, one common strategy is to label every artefact volume with a separate single volume regressor, thus eliminating the effects of artefacts on the model estimation. Another way to avoid these issues is to remove the volumes containing artefacts entirely [9]. This method however results in a concatenated dataset, which introduces special considerations in the subsequent data processing steps, relating to filtering, model specification and brain masking. Conventional temporal high- or low-pass filtering for complete datasets without artefact removal introduces filter edge effects whenever artefacts occur in the time series. Using such filters after concatenation introduces filter edge effects between the concatenated time series segments whenever there is a stepwise intensity change.

The normalization of the brains in a group study to a template brain is another issue that is not straightforward in non-human primates. One important prerequisite is the removal of non-brain tissue from the images, since the anatomical variability of such tissue in primates is high. In NHP studies that use custom coils to maximize signal to noise ratio (SNR), there often exists a strong image intensity gradient. This image intensity gradient combined with the lack of suitable a priori images makes an automated brain extraction difficult.

In this paper, we illustrate the benefits of using a combined approach of trial selection and concatenation, non-standard realignment and custom filtering, as well as an adaptive brain extraction algorithm on two typical NHP datasets and compare this to a more standard approach of modelling the artefacts in a design matrix in SPM.

## Materials and Methods

### Animals

Two male macaque monkeys (M1&M2, Macaca mulatta) were used in the experiment. All procedures were approved by the local authorities (Regierungspraesidium), and were in full compliance with the guidelines of the European Community (EUVD 86/609/EEC) for the care and use of laboratory animals. The surgical procedures are described in detail elsewhere [10].

### Training and Task

We used a mock scanner setup to simulate conditions in the scanner [3]. To limit motion artefacts, special training procedures were implemented to make sure that animal movement within experimental trials was minimal. To this end, motion sensors were used to measure jaw and body movements [3].

The animals were gradually familiarized with the mock scanner chair, the head holding apparatus, the feedback from the monitoring of eye movements and body- and jaw motion, and finally exposed to scanner noise. The animals had to perform sequences of tasks (trials) that consisted of the following components: to remain still and maintain visual fixation before and during the presentation of a sequence of six familiar fractal images; after stimulus presentation, the animals were required to stay still for a period of 8–10 s before reward delivery (a droplet of fruit juice). Trials in which movement was detected by the motion sensors were instantly aborted (for detailed task and trial information see [11]).

### MR system and coil

We performed experiments in a vertical 7-T magnet with a bore diameter of 60 cm (Bruker, BioSpin GmbH, Ettlingen, Germany). We used a whole head volume coil (linear saddle coil) designed by H. Merkle [12].

### Imaging Protocol

For animal M1, we used a 2-segment interleaved T_2_*-weighted gradient-echo echo planar imaging (GE-EPI) sequence. In-plane resolution was 1.2×1.6 mm^2^. Field of View (FOV) was 115×115 mm^2^, echo time (TE) was 19 ms, repetition time (TR) was 1000 ms, intervolume time was 2000 ms, Bandwidth (BW) = 158.7 kHz. We acquired 19 contiguous slices. A single-shot GE-EPI sequence was used for animal M2. In-plane resolution was 0.75×0.75 mm^2^. FOV was 96×96 mm^2^, TR was 1000 ms, intervolume time was 1000 ms, TE was 21 ms, BW was 156.25 kHz. We acquired 11 contiguous slices. For both datasets, slice thickness was 2 mm gapless. Comprehensive shimming was done at the beginning of every session. We acquired functional data in runs of 5 minutes each. More detailed information about the animals, implants, MR system & coils [13], training, task, motion sensors and imaging protocol is provided in [3] and [11].

### Sample Datasets

Each session consisted of several short sequences (runs) of 5 minutes, in each of which 150 (M1) or 300 (M2) functional volumes were acquired containing a mixture of successful trials and artefact periods. To demonstrate the benefits of our algorithm, we used two sample datasets. The first dataset consisted of 4 sessions acquired on different days for animal M1. The second dataset consisted of one full day of scanning for animal M2. These protocols are representative for many NHP fMRI experiments, in which data are collected over several days.

### Overview of methods used in fMRI Sandbox

All processing steps were performed with fMRI Sandbox (FSB, available on Google Code: http://code.google.com/p/fmri-sandbox/ Accessed 2011 Dec 7.), unless otherwise stated. The software has a built-in logging functionality to record all processing steps made by the user. It allows the user to interact with the data in a more direct way than established software toolboxes like SPM or FSL [14], [15]. Moreover, it contains integrated approaches to several problems common to awake NHP fMRI. After FSB preprocessing which used a modified SPM2 function for part of the realignment, further processing was done with SPM5 in all cases.

In the first FSB step, the user can semi-automatically and interactively select trials and volumes that contain artefacts and reject them from the subsequent analysis. We refer to this procedure as data concatenation.

In a second step, we introduce a custom realignment method (2-step realignment) for datasets containing distortions even within the concatenated data for which the trial structure is known. This method we call 2-step realignment.

Another possibility integrated into fMRI Sandbox is a custom temporal filtering algorithm that takes into account the specific properties of concatenated data and bridges the gaps that are created by concatenation before temporal filtering.

The last feature of fMRI Sandbox useful for awake NHP fMRI is an interactive brain extraction algorithm that allows removal of muscle tissue from macaque brains with an interactively generated a-priori mask.

In the following sections, we explain each of these steps in detail. Each step can be used independently of the others, even though some steps (e.g. 2-step realignment) will facilitate the application of other steps.

### Trial selection and data concatenation

**Figure 2:**
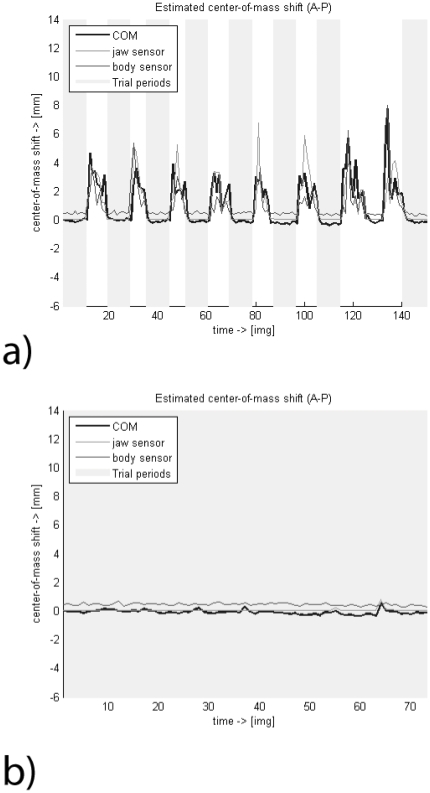
Animal movement and trial concatenation. Relationship of jaw and body movements with instabilities of the fMRI data before a) and after b) trial concatenation of one sample run of animal M1. The black thick line indicates the centre of mass (CoM) shift in millimetres relative to the first volume in the time series (see also [3]), representing apparent brain movement. The thin grey line indicates the recorded signal from the jaw movement sensor, and the thin black line the signal from the body movement sensor. Trial periods are shaded in light grey. Most movement occurs between trials and causes large artefacts visible in the CoM shifts. Trial concatenation removes most volumes containing such artefacts.

For the reasons described in the introduction, subject movement needs to be addressed explicitly in the analysis of NHP fMRI experiments at high field. In particular, even in the absence of head-movement, image distortions can occur due to movements of the body or of limbs, in the following referred to as ‘body movement’ or of the jaw, in the following referred to as ‘jaw movement’. In our representative sample dataset, large body and jaw movements of the animal were most common in between-trial periods. Movement usually occurred after the end of each trial when the animal was rewarded (Figure 2a). Large movements during trials were generally detected by motion sensors, and the trial accordingly aborted. In some cases, however, slight animal body movement was not detected by the sensors and led to image degradation in the form of visible ghosting even within a completed trial. If the movement was only slight and the distortions were not instantly visible, it led to apparent image shifts in the phase encoding direction or to distortions. In rare cases, such minor body movements could also result in serious image degradation (animal M1), making the images unusable for further processing.

In order to obtain a clean time course, the first step was to label trial periods and remove all scan epochs outside of trials (Figure 2b). For the 2-segment GE sequence (animal M1), trial periods were then automatically examined for motion artefacts using fMRI Sandbox. This was done by calculating the ratio of the mean intensity of a predefined area within the brain to the mean intensity of the area outside of the brain that was most affected by ghosting artefacts; thresholds were set manually and the result inspected online. Data were finally inspected by eye. Center of Mass (CoM) shifts were displayed together with the data timecourse to facilitate artefact detection. Where artefacts were detected, data for the whole trial were removed. We thus obtained a temporally discontinuous series of volumes that were devoid of visible artefacts.

Using this semi-automated process with manual input introduces some degree of subjectivity into the trial selection process. However, similar procedures are also used e.g. in EEG data cleaning, and are not suspected to introduce condition-specific biases [16]. An investigation of inter-rater reliability has not been done in the context of our study.

### Realignment within trials

Because of optimized scan parameters [12], [17], and motion control of the animal's jaw and body, distortions within trials were minimal. Nevertheless, due to physiological motion, e.g. breathing, there were some slight shifts of the brain in the Anterior – Posterior (A-P) axis within the trials. A standard realignment algorithm, such as the one implemented in SPM, does not give priority to shifts in the phase direction over all other spatial transformations. As rotations and shifts in any other direction were physically impossible, we only compensated for image shifts in the phase direction. We used a modified SPM2 routine to realign every volume in a trial to its respective first volume, correcting only apparent head motion along the A-P axis. To avoid unnecessary re-slicing and thus interpolation of the data, we only kept the spatial transformation parameters; re-slicing was done at a later stage.

### Realignment and normalization of mean trial images

Our experimental design allowed animals to move between trials. Because of changes in animal position between trials, the apparent position and shape of the brain was not exactly the same over trials. Since using a simple 6-parameter rigid body co-registration as implemented in SPM would not have accounted for distortion of mean trial images, we corrected for brain position shifts and image distortions between trials with a custom algorithm. First, mean images of the different trials were calculated. These were then non-rigidly coregistered to the first mean trial image with algorithms from SPM2 using an affine 12-parameter transformation (rotation, shifts, scaling and shears in x, y and z axes).

### 2-step realignment

To combine in-plane realignment within trials and 12-parameter nonrigid coregistration of mean trial images, we used the following algorithm:

For each functional volume, the individual combination of within- and between-trial realignment and normalization transformations was calculated, and then applied to each single data volume to nonrigidly coregister to the mean volume of the first trial. Images were re-sliced at this point in order to prepare them for further processing.

This combination of realignment within trials and realignment and normalization of mean trial images is in the following referred to as 2-step realignment. A separate paper [18] lays out the details and reports a detailed comparison of this realignment method with a number of other approaches.

### Temporal filtering

**Figure 3:**
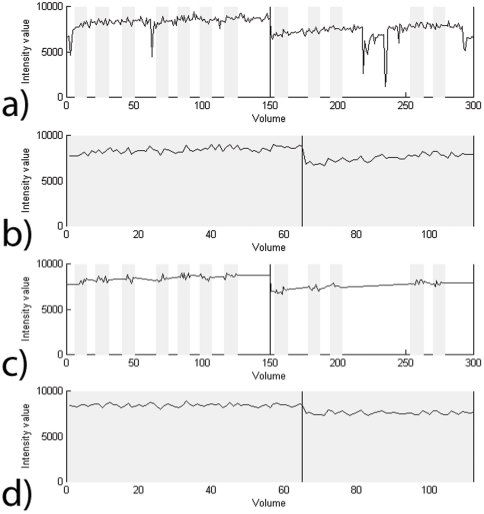
Timecourse of a surface voxel affected by distortion. Effect of the various processing steps on a single voxel time course. The x-axis depicts time in volumes, the y-axis shows voxel intensity values. The voxel time course: a) before any processing; b) after trials have been concatenated, showing low frequency drifts and a stepwise change at the boundary between scanning runs (vertical black line) c) when artefact periods are interpolated between trials in order to reproduce the original temporal arrangement of volumes; d) after filtering of interpolated data and removal of interpolated segments; there is now less variance in the time course due to filtering, though a step between runs remains.

Intensity drifts and stepwise shifts in voxel intensity remained even after trial concatenation, artefact removal and realignment (Figure 3a, b). In order to remove these and other general scanner drifts and noise from the voxel time courses, a high-pass filter with a cut-off of 96 s was used. We linearly interpolated artefact periods in every run separately (Figure 3c) and then filtered the single runs one by one. For interpolation, the voxel-wise average of the last two volumes of every trial was calculated, likewise the average of the first two volumes of the subsequent trial. We linearly interpolated the values of these two averages over the time elapsing between them in the actual scan.

After the filtering was done, the interpolated data were discarded, and the trials concatenated. This way, we obtained temporally filtered time courses from concatenated data (Figure 3d). What still remained were stepwise intensity differences between runs that had been filtered. To eliminate the effects of such intensity changes, we modelled every run separately as a session in SPM.

### Brain extraction

Conventional brain extraction is usually done by combining an a-priori intensity distribution image with the intensity thresholded image that needs to be extracted. Such algorithms failed to extract the brain from the surrounding tissue in the functional volumes in our study. This happened for two main reasons. First, a suitable probabilistic map of the distribution of brain and tissue for macaque brains was not available for the functional EPI images. Second, given the severe distortions that we observed at 7T, using static probabilistic distributions did not improve the brain extraction for EPI images because of the distortion of the latter at high field. Even though a number of probabilistic atlases are available, some were created for a different primate species [19]–[22], making their use less suitable for our sample of *Macaca mulatta* scans. One atlas [23] comes with a T2-weighted average template of a *Macaca mulatta* brain and recently, another template has been made available that aims to integrate the two monkey species *M. mulatta* and *M. fascicularis* [24]. Still, using any of these templates was not sufficient to successfully extract the brain from the surrounding tissue in our samples.

**Figure 4:**
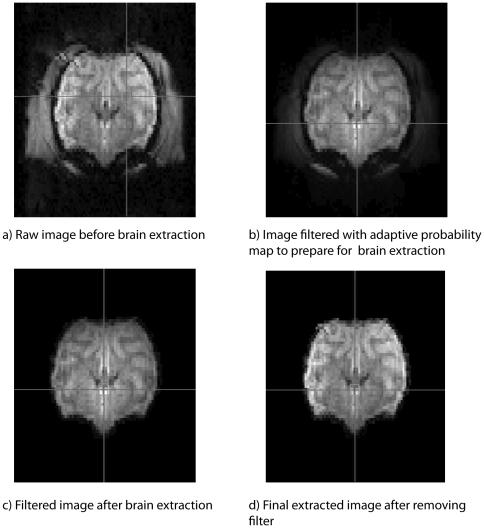
Adaptive brain extraction algorithm. Demonstration of the effects of the algorithm implemented in fMRI Sandbox on an example slice (#10) of the mean image of a sample dataset M1: a) before applying any mask or filter; b) after applying a filter to suppress matter outside the brain; c) after brain extraction, now containing intensity changes in the brain due to the prior filtering; d) after a mask with the extraction data derived from c) has been applied to the original dataset.

Using conventional probabilistic maps created for human imaging was not possible because of different size and tissue distribution. Furthermore, B_1_ inhomogeneity is a common problem at 7 T. As is typical for high-field NHP fMRI experiments, our scans were acquired with custom made Radio Frequency (RF) coils, which were relatively small in order to increase the SNR and needed to be open in order to fit our visual stimulation system. The high field in combination with the custom coils did not deliver the kind of B_1_-homogeneity common in lower-field scanners and standard clinical coils used in human fMRI studies. This caused intensity gradients in the images which made a purely automatic detection of brain boundaries difficult. As software like FSL, MRIcro and SPM did not achieve a clear extraction of brain tissue from surrounding tissue, we created our own brain extraction routine with additional functionality. Our algorithm improves brain extraction by allowing the user to interactively determine the brain border and extract only brain without including other tissue such as muscle or eyes. The results are illustrated for monkey M1 in Figure 4.

In Figure 4a, one slice of the raw image before brain extraction is shown. Image intensity of the brain and of adjacent tissue is similar, which makes a segmentation based on image intensity differences alone difficult. To address this issue, a filter which increases the intensity in the middle of the image and decreases intensity in the periphery of the image is applied in Figure 4b. This results in an image intensity difference between the brain and other tissue, which allows for better brain extraction. The strength of the filter can be adjusted by the user to maximize image intensity differences between tissue and brain for the processed dataset. Additionally, the centre of the filter (crosshair) can be positioned such that the intensity differences for the image under observation are optimized for the specific dataset and region of interest. This filtered image is then intensity thresholded. The thresholded image is used to create a binary mask, which is then filled from inside to determine the brain border. In a next step, this mask is multiplied with the filtered image to generate a preview of the actual brain extraction. The user can interactively optimize brain tissue selection with sliders for the filter and the intensity threshold used. (Figure 4c). Once the optimal combination of bias field and extraction mask is found, the extraction mask can be applied to the original volume, which results in Figure 4d. Thus, the bias field is not included in the further analysis. The whole procedure is semi-automated and is usually completed within less than a minute with good separation of brain and other tissue.

As our brain extraction algorithm works on the mean image of all volumes to be processed, prior realignment was necessary at this step. Without realignment, a mean image of all volumes would have included either not all of the brain or also tissue of no interest, e.g. the large jaw muscle in some of the volumes.

### Comparison of sample data analysis with conventional algorithm and new algorithm

In order to evaluate our non-standard methods with the established practice for human subjects, we ran 5 different analyses (Methods 1–5) on two datasets. To assess each method, we calculated the contrast between the fixation and the stimulation phase of the trial.

Two of these analyses were done with standard SPM preprocessing, with and without artefact modelling, while the remaining three cumulatively applied our NHP-specific preprocessing methods. Regressors were modelled before trial concatenation with standard SPM functions (event durations convolved with a canonical hemodynamic response function) to accurately represent the expected hemodynamic response to the stimulus presented. Any fMRI data concatenation was applied to the regressors as well to maintain temporal coherence.

As our experimental setup involved frequent short scan periods of 5 minutes (runs), often containing a small number of successful trials, run effects were modelled as session effects. Modelling single trials as sessions is possible but not suitable for designs that aim to elucidate differences between two or more trial types, since both trial types would be scaled differently; thus we avoided it here.

**Figure 5:**
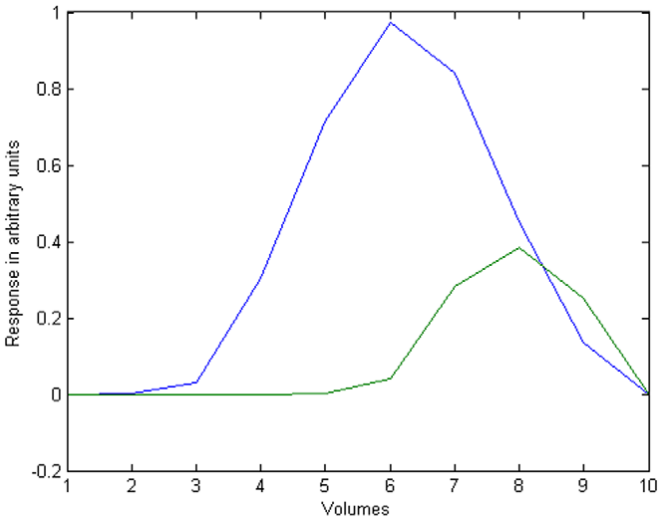
Hemodynamic regressors. Modelled fixation and stimulation regressors for one single trial. The blue line depicts the modelled regressor for pure fixation (6 s), the green line the modelled regressor for visual stimulation (2 s).

Figure 5 shows example fixation and stimulation regressors for a single trial.

Except for Method 1, the same trials were used or excluded for all methods. All detected artefacts and trials affected by artefacts were labelled for Method 2, while all detected artefacts and trials affected by artefacts were removed for Methods 3–5.

For animal M1, we excluded 9 of 62 trials or 15% of all trials, as well as 434 of 1800 volumes (24%) altogether. Our automatic detection algorithm correctly identified 431/1800 volumes (24%) and 6/62 trials (10%) as affected by artefacts. Upon closer manual inspection, we rejected or labelled another 3 trials from the dataset. These contained visible artefacts that were not automatically detected by our detection algorithm. We also rejected or labelled all intertrial periods. We note that signal from animal movement sensors for the rejected trials was not sufficiently different from the non-rejected trials to lead to automatic trial abortion during the experiment based on the sensor thresholds used. Setting the automatic detection threshold lower would have been another option, but at the risk of increasing false positives and reducing the number of successful trials.

For animal M2, automated artefact detection was not possible, since it was a single-shot acquisition and segmentation artefacts could not be observed. Thus, we only discarded inter-trial periods, retaining all trial data for analysis.

Smoothing was done with a FWHM (Full Width at Half Maximum) kernel and a spatial extent of 2×2×2 mm^3^ with SPM5.

In the SPM model specification, a high-pass filter with a cut-off of 128 s was used for all analyses, including the ones that had been filtered within fMRI Sandbox before. All datasets were AR(1) corrected. Grand mean scaling was used rather than global scaling.

For quantitative analysis we thresholded the resulting SPM t-maps at T = 3.11 (p<0.001 uncorrected) and counted the suprathreshold voxels for each method and both animals.

#### Method 1: Standard modelling

We took the original raw datasets and analyzed them in a standard way, running all volumes through standard SPM re-alignment and re-slicing, and model setup with our hemodynamic regressors as our task regressors for fixation and stimulation. We did not use motion regressors, since real head movement was not possible.

#### Method 2: Standard modelling with artefact regressors

In order to get a measure of the best possible approach for a standard SPM analysis, we removed the influence of artefacts on the statistical map. To this end, an additional artefact regressor was modelled separately for every artefact-affected volume, thus effectively removing artefact-tainted variance from the dataset. For M1, this included every visible artefact volume, plus every single volume in all trials containing any artefacts. Since artefact volumes could not be individually identified for animal M2 (see above), we simply modelled all volumes outside of trials as artefacts.

#### Method 3: Concatenated data

To determine the contribution of different processing steps, we ran the steps of our algorithm separately. As a first step, inter-trial periods as well as artefact-tainted trials were directly removed from the dataset before further processing. For all concatenated datasets, artefact labelling regressors are not included any more, since all detected artefacts were already removed by the concatenation.

#### Method 4: Concatenated data with 2-step realignment

As a second step to elucidate the effects of our algorithm, we used our 2-step realignment procedure on both datasets.

#### Method 5: Concatenated data with 2-step realignment and high-pass filtering

As our next step aimed to remove linear and nonlinear trends as well as high-frequency noise from the dataset, all voxels were separately high-pass filtered with a filter cut-off 96 s after linearly interpolating out-of-trial and bad trial time points.

## Results

### Brain extraction

**Figure 6:**
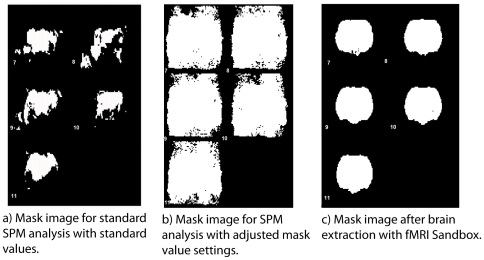
Mask images for animal M1. a) A mask from a standard analysis with artefact regressors. Due to the strong intensity changes in many voxels over time, the mask image is seriously degraded. b) Mask image after the mask threshold value has been adjusted to include the whole brain. The mask does now cover not only the brain but all other tissue as well. c) A mask image resulting from running a standard analysis after our advanced preprocessing. The mask image now homogenously covers the whole brain. Slices 7–11 are depicted.

For M1, the performance of our brain extraction algorithm is illustrated in Figure 6, comparing SPM analysis masks derived from different brain extraction approaches. In Figure 6a, the mask for the non-concatenated data of a standard analysis, including artefact regressors for each separate artefact volume, is very fragmented due to automatic thresholding done by SPM with the default mask value. In Figure 6b, the mask threshold value has been sufficiently lowered to include all of the brain, but also includes other tissue and even empty space around the brain. Figure 6c shows the resulting mask after semi-automated brain extraction in fMRI Sandbox. Areas of no interest around the brain are no longer included; at the same time, nearly the whole brain is successfully extracted.

For M1, analysis methods were compared using fMRI Sandbox brain extraction as illustrated in Figure 6c. This excludes Method 1, which was a baseline SPM analysis excluding all fMRI Sandbox functionality. For M2, however, brain extraction failed for Methods 1–3, since SPM's standard realignment methods were not adequate for this data set. For a fair comparison of the five methods on M2 data, accordingly, no brain extraction was employed with any method.

### Comparison of sample data analysis with conventional algorithm and new algorithm

**Figure 7:**
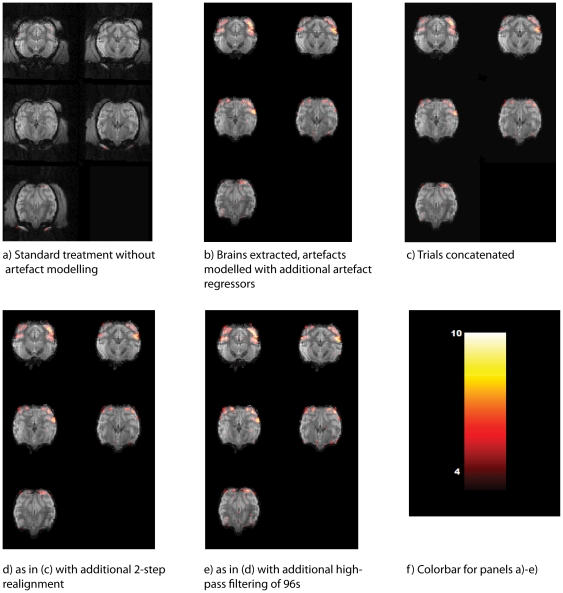
Activation maps for animal M1 after the consecutive data processing steps. All runs have been modelled separately. Activation maps show the contrast for visual stimulation vs. simple fixation, overlaid on 5 slices (7–11) of the mean EPI-images. Colour bars show T-values; voxel threshold p = 0.001 uncorrected (T = 3.11); all cluster sizes shown.

**Figure 8:**
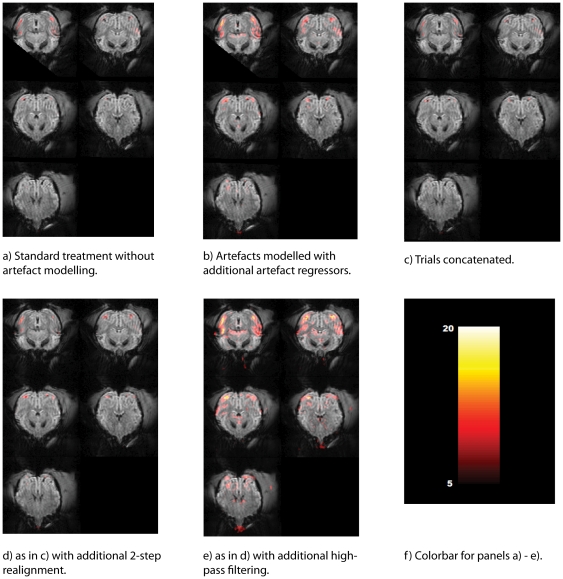
Activation maps for animal M2 after the consecutive data processing steps. All runs have been modelled separately. Activation maps show the contrast for visual stimulation vs. simple fixation, overlaid on 5 slices (2–6) of the mean EPI-images. Colour bars show T-values; voxel threshold p = 0.05 FWE corrected (T = 4.96), all cluster sizes shown.

**Figure 9:**
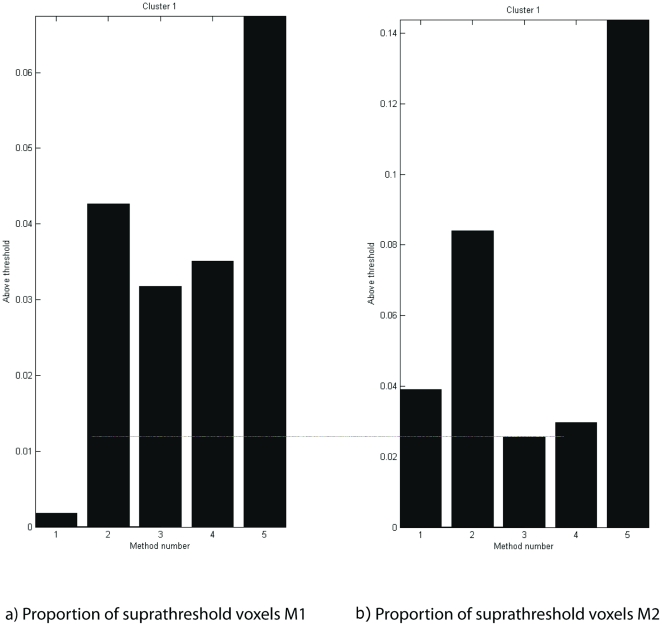
Proportion of suprathreshold voxels for animals M1 & M2. Number of voxels above a threshold of T = 3.11 are shown for methods 1–5 are depicted. a) Animal M1. b) Animal M2. Method 1: Standard modelling with SPM realignment; Method 2: Standard modelling with SPM realignment and artefact regressors; Method 3: Concatenated data with SPM realignment. Method 4: Concatenated data with 2-step realignment; Method 5: Concatenated data with 2-step realignment and high-pass filtering within FSB.

In Figures 7 and 8 we show the same slices of our sample datasets (Figure 7, M1; Figure 8, M2) processed with different methods. The figure shows results for the contrast stimulation vs. fixation in a fixed-effects model. For each animal, Figure 9 shows proportions of suprathreshold voxels for this contrast, separately for each method and animal.

#### Method 1: Standard modelling

Figures 7a and 8a show the maps resulting from a standard SPM analysis without explicit modelling of artefact volumes. The proportion of suprathreshold voxels for animal M1 is very low, not even reaching 1% of the whole dataset (Figure 9). For M2, the corresponding proportion is nearly 4%.

#### Method 2: Standard modelling with artefact regressors

Here, the same dataset without concatenation was used, but the artefact volumes in the dataset were labelled with separate regressors. For M1, statistical maps were improved (Figure 7b). Both for animal M1 and M2, the proportion of suprathreshold voxels was higher than for method 1, above 4% for animal M1 and above 8% for animal M2.

#### Method 3: Concatenated data

Figures 7c/8c show the maps for the concatenated dataset, where inter-trial periods as well as artefact-tainted trials were removed during preprocessing. For animal M1, at first glance, functional maps look very similar, yet the quantitative measures (Figure 9) were slightly worse than for the artefact modelling. Still, there was a clear improvement compared to the original analysis without artefact modelling with more than 3% of all voxels in the dataset reaching suprathreshold level for M1. For M2, method 3 produced a smaller proportion of suprathreshold voxels than method 1.

#### Method 4: Concatenated data with 2-step realignment

In Figure 7d/8d, we used our 2-step realignment algorithm, which for animal M1 did not visibly affect the map. The quantitative measure (Figure 9) improved slightly compared to the concatenated data realigned with the standard SPM function, yet were still clearly worse than for method 2. For M2, similarly, methods 3 and 4 produced rather similar results.

#### Method 5: Concatenated data with 2-step realignment and high-pass filtering

In order to remove linear and nonlinear trends from the dataset, all voxels were separately high-pass filtered after linearly interpolating out-of-trial and bad trial voxel time points (Figures 7e/8e). For both animals, this increased the proportion of suprathreshold voxels above the results obtained with the SPM best practice method 2.

## Discussion

As awake NHP scanning at high magnetic field is becoming more widespread, and new issues with movement artefacts are arising due to the high susceptibility of the field homogeneity to animal motion, there is an urgent need for new approaches for movement artefact handling, experimental design and data analyses. In this paper, we propose special-purpose refinements and additions to data analysis that address several issues specific to NHP fMRI experiments at high fields. All of the methods presented here are part of fMRI Sandbox, a toolbox to interactively process fMRI data, which can be downloaded from Google Code: (http://code.google.com/p/fmri-sandbox/). It allows more efficient use of acquired animal data, thereby reducing the number of scans needed. Elements of this approach have been laid out in earlier work [3], [11], [18].

Our approach relies on the trial-based training and data acquisition approach introduced in [3] to minimize animal motion during experiment periods of a scan and uses elements of the data processing strategies introduced by [9]. We extend this approach by introducing specific realignment strategies that focus on in-plane shifts; moreover, we compensate for brain distortions within and between runs and sessions with a non-rigid coregistration algorithm [18]. Our temporal filtering strategy addresses the issue of discontinuous volume time series and global and local intensity changes over single and multiple runs. It furthermore allows the use of established fMRI analysis software packages (e.g. SPM) for further processing.

Our approach deviates from the conventional approach to human data analysis in several ways. Firstly, we do not rely on complete datasets for analysis, as usually done in human scanning. Other artefact treatment algorithms have been proposed [25], but these do not allow for completely interactive handling of series of functional scans to the extent required for the processing of our datasets here. Awake monkey imaging at high field is different from human imaging in many respects. In our study, we did not have actual head movements, but field changes due to whole body movement that usually occurred at predictable times and lasted for several volumes. Even in an optimal case, less than half of the volumes of a functional scan contain data which are related to an experimental task. Trial periods last between 10 and 20 seconds, while the subsequent reward-associated movement takes up to 20 seconds before another trial is initiated by the animal. Additionally, the animal does not always finish every trial, resulting in further unusable volumes. A simple interpolation of artefact volumes with data from volumes before and after such artefact periods would not really be useful. We therefore decided to completely eliminate such volumes, which brought the issue of concatenated datasets about. In principle, it is not appropriate to process such datasets in a conventional way, as the assumption of temporal coherence of volume data does not hold. In order to circumvent this, we used a custom interpolation and filtering method. After preprocessing is done, the dataset can be treated as a conventional dataset, and filtering will not have additional adverse effects.

We removed whole trials instead of single artefact volumes, because cutting out single volumes from a time series brings about two issues. First, time discontinuities are introduced again within the time series. Secondly, it remains possible that the animal movement that produced image deterioration in one volume also led to field changes within the same trial. To avoid this, we have chosen not to include the whole trial if affected by artefacts.

To show the improvement possible with our methods, we began with two established approaches on two sample datasets acquired with different sequences and animals. The first was to simply run the SPM estimation on the whole dataset without explicitly labelling volumes containing artefacts. This would be the standard approach taken if manual inspection of datasets would be too time-consuming or automated inspection of datasets would not be available or established.

As an alternative best-practice standard approach, we used a tailored method where artefact regressors were used to remove the effect of artefact volumes. For M1, we modelled all volumes with visible artefacts, plus all additional volumes of trials where artefacts occurred. For M2, where artefacts were generally not visible, we simply added artefact regressors for all inter-trial volumes. For M1, this approach yielded a much better and more localized map with a much higher proportion of voxels in the dataset reaching statistical significance. For animal M2, likewise, the number of suprathreshold voxels strongly increased in comparison to the standard approach.

For the next step, we produced a pruned dataset consisting only of trial periods. This step reduced the number of suprathreshold voxels for both animals. This was likely due to filter edge effects between the concatenated trials because of the stepwise changes at trial boundaries and a reduction in the effective degrees of freedom due to the removal of a large number of volumes from the dataset.

To account for position changes within trials and distortions and local intensity changes between trials we used a special 2-step realignment procedure. The realignment procedure consisted of a within-trial rigid and a between-trial non-rigid coregistration of volumes [18]. We observed a higher number of suprathreshold voxels for both animals. In M2, furthermore, our 2-step realignment procedure was essential to allow successful brain extraction, though to maintain comparability of methods, we have not reported its effects here.

In a final step, we temporally filtered the data after having interpolated the non trial periods in order to preserve temporal coherence of the dataset. This yielded a clear advantage for our combined method in comparison to all other methods in respect to the number of suprathreshold voxels. This is likely due to the removal of stepwise changes at trial boundaries in the preprocessing of the data with the high-pass filtering of interpolated data as well as the filter setting we are using for the high-pass filter. To explore if only the filter setting was responsible for the improvement, we tested different filter settings both for the SPM analysis as well as for our interpolated filtering, but found no clear effect of filter size setting.

Issues of image distortion can also be addressed by improvements in signal acquisition. Phased array coils [26] have recently been used for a number of studies [27]–[31] with parallel imaging [32] and sequences like GRAPPA [33] or SENSE [34] can reduce distortion, although the reduced distortion comes at the expense of SNR [32] and may decrease BOLD MRI sensitivity in areas unaffected by artefacts [35]. For a review of these techniques, see e.g. [36]. Our approach is complementary to these, providing an optimal data analysis route even for data acquired with either surface or standard extremity coils without parallel imaging (as in the vast majority of awake monkey fMRI studies).

Multi-shot imaging [37] has likewise been successfully used to reduce image distortion at high field, in awake animals [11], [17], [38], and in anaesthetized preparations with up to 16 segments [38]–[40]. Still, given the short trial durations that monkeys can be trained to tolerate without reward, using a multi-shot imaging sequence with more than 2 shots (like one of the sample datasets used here) reduces the efficiency by increasing the intervolume time, and thus considerably reducing the number of samples within a trial.

Another approach to address distortion is based on the acquisition of B_0_ field maps, to correct for distortion [6]–[8]. Because of the susceptibility of the magnetic field to animal movement and position changes, the field was frequently changed between trials in our experiments. To use the field map approach in our study would have necessitated the acquisition of a field map for each single trial or the use of dynamic field maps acquired for each single volume [41]. Another option would have been to do field map correction for single sessions to account for the static field map distortion component only [12]. We have not explored any of these options here.

Navigator echoes can also be used to correct movement [42], segmentation artefacts [37] breathing effects [43], or geometric distortions [44] in fMRI data. However, the use of navigator echoes and shim navigators for awake monkey imaging at high field was found to be inferior to both standard 3D and custom 2D realignment methods for the removal of motion in the time series [45].

Even though some of the issues we address have been addressed earlier with other methods, our proposed processing algorithm is complementary to these methods mentioned above, and does not require additional investments in hardware, software or sequence programming.

The notion of combining animal training with motion-controlled trials in the scanner paves the way for the analysis algorithms we outline here. Our approach goes beyond the earlier attempts by introducing analysis methods that allow scavenging useful data from artefact-tainted datasets. This is especially relevant for high-field studies in which the probability of obtaining artefact-tainted data is high. By using advanced animal training, scanning and analysis methods, it was possible to select trial periods devoid of motion artefacts and combine them into new datasets. Processing these datasets with the algorithm laid out in the methods section markedly improved the quality of the datasets which could then be analyzed in a conventional way. In another paper we have demonstrated the extension of the single subject analysis methods outlined in this study to group datasets [11].

Because high-field scanning in general is very susceptible to subject motion, the methods described here could also be considered for application in human scanning. One major difference between the conventional data acquisition method used so far in human scanning and the approach taken here lies in the structuring of the scanning time into separate trials that can be separately selected for further processing or discarded based on the artefact content. In an application to patient scanning in clinical studies, it would e.g. be possible to instruct participants to lie still for a few seconds and allow small movements for a few seconds after each trial, emulating the experiment structure proposed for the acquisition of animal data. As in the present work, motion contaminated scan periods could be interpolated and conventional temporal filtering methods applied before removing these scan periods again to avoid introducing artificial data points. In this way, high field imaging may become possible for studies with patients, children or nervous participants who find it impossible to remain completely still for the whole duration of the scans.
